# Quality of care and quality of life of people with dementia living at green care farms: a cross-sectional study

**DOI:** 10.1186/s12877-017-0550-0

**Published:** 2017-07-19

**Authors:** Bram de Boer, Jan P.H. Hamers, Sandra M.G. Zwakhalen, Frans E.S. Tan, Hilde Verbeek

**Affiliations:** 1 Department of Health Services Research, Faculty of Health, Medicine and Life Sciences, CAPHRI Care and Public Health Research Institute, Maastricht University, Duboisdomein 30, 6229 GT Maastricht, The Netherlands; 2 Department of Methodology and Statistics, Faculty of Health, Medicine and Life Sciences, CAPHRI Care and Public Health Research Institute, Maastricht University, Maastricht, The Netherlands

**Keywords:** Dementia, Long-term care, Nursing homes, Quality of care, Quality of life, Small-scale living facilities

## Abstract

**Background:**

Many countries are introducing smaller, more home-like care facilities that represent a radically new approach to nursing home care for people with dementia. The green care farm is a new type of nursing home developed in the Netherlands. The goal of this study was to compare quality of care, quality of life and related outcomes in green care farms, regular small-scale living facilities and traditional nursing homes for people with dementia.

**Methods:**

A cross-sectional design was used. Three types of nursing homes were included: (1) green care farms; (2) regular small-scale living facilities; (3) traditional nursing homes. All participating nursing homes were non-profit, collectively funded nursing homes in the south of the Netherlands. One hundred and fifteen residents with a formal diagnosis of dementia were included in the study. Data on quality of care was gathered and consisted of outcome indicators (e.g. falling incidents, pressure ulcers), structure indicators (e.g. hours per resident per day), and process indicators (e.g. presence, accessibility and content of protocols on care delivery). Furthermore, questionnaires on cognition, dependence in activities of daily living, quality of life, social engagement, neuropsychiatric symptoms, agitation, and depression were used.

**Results:**

Data showed that quality of care was comparable across settings. No large differences were found on clinical outcome measures, hours per resident per day, or process indicators. Higher quality of life scores were reported for residents of green care farms in comparison with residents of traditional nursing homes. They scored significantly higher on the Quality of Life – Alzheimer’s disease Scale (*p* < 0.05, ES = 0.8) indicating a better quality of life. In addition, residents of green care farms scored higher on three quality of life domains of the Qualidem: positive affect, social relations and having something to do (*p* < 0.05, ES > 0.7). No differences with regular small-scale living facilities were found.

**Conclusions:**

Green care farms seem to be a valuable alternative to existing nursing homes. This is important as people with dementia are a heterogeneous group with varying needs. In order to provide tailored care there also is a need for a variety of living environments.

## Background

The number of people with dementia is expected to double every 20 years reaching almost 75 million by 2030 worldwide [1]. A substantial proportion of people with dementia live in nursing homes because they require complex care that cannot be provided in the home situation. There is considerable debate about the quality of nursing home care for people with dementia. The care at traditional nursing homes often focusses on physical care, keeping residents safe, and preventing health care problems (e.g. [2]). However, in recent years there has been more emphasis on a psychosocial and more homelike care concept with an increased interest in values such as quality of life, autonomy and striving to allow residents of nursing homes to continue the life they had before admission as much as possible [3, 4]. This change in care concept can also be seen in policies, strategies or frameworks launched in many countries aimed at improving the quality of care and quality of life for people with dementia living in nursing homes.

In many countries existing nursing homes are changing, and new initiatives are developing that redesign nursing home care with the aim to better meet the needs of people with dementia and to improve the quality of care and quality of life of nursing home residents, focusing on small-scale, homelike care facilities [3]. These facilities follow a psychosocial approach of care that emphasizes, normalization, quality of life and person centred care. Provision of care is organized around small groups (approximately 8 residents); residents and staff form a household together, so daily activities (cooking, cleaning, etc.) are integrated with daily care. Some studies have found that there are benefits to such small-scale, household-like facilities, such as better nutritional status, more engagement in activities and a better quality of life [5, 6]. However, others have reported no differences in the quality of life of residents of small, home-like facilities and residents of traditional nursing homes [7, 8]. Some studies suggest negative effects such as more behavioural problems (e.g. [9]). Although there have been some studies (e.g. [10, 11]), the need for more comparative research on the effects of small-scale facilities was highlighted in a recent literature review [12].

The green care farm is a new type of small-scale, homelike long-term care facility that has been developed in the Netherlands over the last few years. Green care farms combine agricultural with care activities and are aimed at involving residents in activities such as gardening, taking care of animals, household chores, and other types of activities that are incorporated into normal daily life [13]. In the Netherlands, there are over 1000 green care farms for a variety of client groups (e.g. people with psychological problems, learning disabilities, or addiction problems). Approximately 200 provide care for people with dementia. There are a few green care farms that provide 24-h nursing home care for people with dementia*.* Green care farms stem from developments within the agricultural sector and provide unique physical and organizational environments for dementia care. Residents of green care farms have free access to outdoor areas and are often exposed to outdoor stimuli (daylight, animals). The physical environment offers many possibilities for both indoor and outdoor activities such as preparing dinner, playing games, picking eggs, gardening, and feeding the animals. Activities can be performed at different areas on the farm, which invites residents to be physically active. Furthermore, because these activities fit within normal daily life, this is considerably different with more traditional nursing homes, where activities often have an institutional character (memory training, bingo) [14]. In addition, compared with other facilities (both large- and small-scale) the way a certain care philosophy is implemented differs considerably. At green care farms, farmers are personally involved and motivated to transfer their vision regarding person centeredness, creativity and radically redesigning dementia care to their staff. They thus often have a different leadership role than managers in existing nursing homes [14].

Research on the effects of green care farms is scarce, especially research on 24-h nursing home care [14, 15]. The first study of 24-h care in green care farms indicated that there are differences between the daily lives of residents at green care farms and traditional nursing homes. Residents of green care farms took part in more activities, enjoyed more social interaction and more time spend outdoors than residents of traditional nursing homes [16].

More research is needed into the differences between green care farms and existing nursing homes with respect to quality of care, quality of life and related outcomes for people with dementia. Therefore the current study investigates two research questions:

1. How does quality of care for people with dementia differ between green care farms, regular small-scale living facilities, and traditional nursing homes?
2. How do quality of life and related outcomes for people with dementia differ between green care farms, regular small-scale living facilities, and traditional nursing homes?

## Methods

### Design

The current study is part of a larger research project of which the protocol is published elsewhere [15]. This was a cross-sectional study, comparing quality of care, quality of life and related outcomes in residents living in green care farms, regular small-scale living facilities and traditional nursing homes. Data were collected between April and October 2014.

### Setting

The data for this study were collected in non-profit, collectively funded nursing homes in the south of the Netherlands. At green care farms both care and agricultural activities are important and the exterior physical environment is different from that of other types of nursing homes. It includes animals, stables, vegetable gardens and other features of a farm environment. Indoor and outdoor activities are incorporated into normal daily life to make it easier for residents to participate. Green care farms and regular small-scale living facilities have approximately six to eight residents and staff forms a household with the residents. There is a steady team of caregivers which have integrated tasks. Daily living is mainly determined by the residents and their informal caregivers and the physical environment is designed to be like an ordinary home environment as much as possible. Regular small-scale living facilities may be situated at the terrain of a larger nursing home or exist as stand-alone facilities in a neighbourhood [3, 15]. Traditional nursing homes have at least 20 residents per ward; caregivers have differentiated tasks and daily life is mainly determined by routines and rules of the organisation.

### Participants

Residents were eligible to participate in the study if their medical record included a formal diagnosis of dementia. Legal representatives were asked to provide written informed consent for participation in the study. Residents were asked to assent, which is defined as a verbal agreement to participate or a non-verbal indication of willingness to cooperate with the study. In order to increase comparability between residents in terms of cognitive and functional status, a matching procedure was conducted two weeks before the measurement. Residents of all participating locations were screened and residents of the traditional nursing homes were selected based on their screening scores in order to match with residents of green care farms and small-scale living facilities [15].

### Measures

All data was collected by a small trained team of researchers and research assistants under supervision of the whole research team. Standardized operating procedures that described how the data had to be collected were used.

#### Quality of care

Quality of care was assessed by means of outcome, structure, and process indicators [17]. The outcome indicators were collected in line with the international prevalence measurement of care problems [18]. Indicators that were collected were falling incidents during the last 30 days; pressure ulcers; malnutrition during the last year; use of psychotropic drugs; and use of physical restraints during the last 30 days. Structure indicators included information regarding the hours worked per resident per day (HPRD); and the educational level of caregivers. The process indicators were the presence, accessibility, and content of protocols about care delivery. More specific, corresponding to themes investigated by the Health Care Inspectorate (IGZ) protocols regarding 5 topics were explored: quality improvement, staff deployment, client records, physical restraints, and medication safety. These protocols were gathered by logging into electronic portals where the up to date protocols could be accessed. Outcome indicators were gathered on resident level, structure and process outcomes were gathered on ward level.

#### Quality of life and related outcomes

Quality of life was measured using two widely used dementia-specific questionnaires: the Quality of Life-Alzheimer’s Disease scale (QoL-AD) [19] and the QUALIDEM [20]. The QoL-AD uses 13 items rated on a four-point Likert scale to assess current quality of life. The QoL-AD consists of 13 items that are rated on a four-point Likert scale; total scores range from 13 to 52 and higher scores indicate better quality of life. Differences of three or more points in total QoL-AD score are considered meaningful [21]. We used both proxy and self-report QoL-AD scores. The Qualidem consists of 37 items about the last 7 days rated on a four-point Likert scale. Items are divided into nine subscales (care relationship, positive affect, negative affect, restless tense behavior, positive self-image, social relations, social isolation, feeling at home and having something to do). Both questionnaires have acceptable psychometric properties [20, 22].

The Revised Index for Social Engagement (RISE) was used to measure social engagement. The RISE consists of 6 dichotomous items that measure positive features of long-term care residents’social behavior in the last 7 days. Scores range from 0 (minimal social engagement) to 6 (maximal social engagement). The RISE has a Cronbach’s alpha coefficient of .73, and an intra-class coefficient of .75 [23].

The Neuropsychiatric Inventory – Nursing Home version (NPI-NH) [24] was used to measure behavioral symptoms during the last month. It includes 12 neuropsychiatric symptoms (delusions, hallucinations, agitation, depression/dysphoria, anxiety, euphoria/elation, apathy/indifference, disinhibition, irritability/lability, aberrant motor behavior, nighttime disturbances, and appetite/eating change). First, the presence of the symptoms is scored (yes/no). Second, the frequency of the symptom is scored using a four-point scale: rarely (1), sometimes (2), often (3) or very often (4). Third, the severity of the symptom is scored as mild (1), moderate (2), or severe (3). Domain scores are calculated by multiplying the frequency and severity scores [24]. The NPI-NH was reported to have a Cronbach’s alpha of .67 and convergent and discriminant validity [24].

Agitation was measured using the Cohen Mansfield Agitation Inventory (CMAI), which captures the frequency of 29 agitated behaviors during the last 2 weeks using a seven-point Likert scale ranging from 1 = never to 7 = several times an hour. CMAI scores range from 29 to 203, with higher scores indicating more agitated behavior [25]. Acceptable psychometric properties were reported for the CMAI [26].

The Cornell Scale for Depression (CSDD) [27] was used to assess signs and symptoms of depression during the last 7 days. It consists of 19 items on five domains: mood related signs, behavioral disturbance, physical signs, cyclic functions and ideational disturbance. All items are rated for severity using a three-point scale (0 = absent; 1 = mild or intermittent; 2 = severe) and items scores are summed. CSDD scores range from 0 to 38 and higher scores indicate more depressive symptoms. The CSDD was found to be valid and reliable [28].

#### Background characteristics

Information on residents’age, gender, cognition and independence in activities of daily living was collected. Age and gender data were retrieved from medical records. Cognitive impairment at the time of the study was assessed with the Standardized Mini-Mental State Examination (S-MMSE) [29]. S-MMSE scores range from 0 to 30, with higher scores indicating better cognition. The Barthel index was used to measure the current independence in activities of daily life [30]. Barthel index scores range from 0 to 20 and higher scores indicate greater independence in ADL.

### Analyses

First, descriptive statistics were used to explore potential differences between green care farms, traditional nursing homes, and regular small-scale living facilities with respect to background characteristics, quality of care outcomes, quality of life and related outcomes. Differences in background characteristics were then assessed using an ANOVA.

Different analysis strategies were used to analyze the quality of care outcomes. First, Fisher’s exact tests were used to test for differences in outcome indicators between green care farms and the other types of nursing homes. The outcome indicators were the dependent variable, and the type of nursing home was the independent variable. Due to the low prevalence or absence of the outcome indicators in some of the nursing home types, or the sampling distribution not being Chi-square distributed, it was not possible to use a logistic regression or chi-square test. Missing values were random, and treated as such. Second, regarding the structure indicators of quality of care, a descriptive approach was used. For each participating nursing homes the average hours per resident per day (HPRD) was calculated, this was also done per educational level, the nightshift was not included in the comparison. Third, the process indicators of quality of care were subjected to a document analysis; the procedure entailed finding, selecting, appraising and synthesizing the data contained in the documents through skim-reading (superficial examination), reading (thorough examination) and interpretation. This iterative process combined elements of content analysis and thematic analyses. Protocols were explored on the following topics: presence, accessibility, and content. The document analysis was not aimed at adjudicating the protocols; rather it had explorative purposes to indicate whether differences between green care farms, traditional nursing homes and regular small-scale living facilities could be found. The document analysis was performed by the first author (BdB) and independently checked by the last author (HV). Differences were discussed in order to reach agreement. Furthermore, findings were discussed within the whole research team. Regarding the outcomes on quality of life and related outcomes, a two-level multilevel regression analysis was carried out, with the QoL-AD, Qualidem, RISE, NPI-NH, CMAI or Cornell as dependent variables, and the type of nursing home as the main independent variable. There was controlled for age, gender, cognition (S-MMSE), and independence in activities of daily living (Barthel index). The residents (level 1) were nested in nursing homes (level 2).

## Results

### Background characteristics

Legal representatives of 115 of 158 eligible residents (73%) provided informed consent for participation in the study. In total, 18 nursing home locations participated in the study, five green care farms, nine regular small-scale living facilities, and four traditional nursing homes. Table 1 provides information on the characteristics of the residents. The ANOVA did not reveal any significant group differences on background characteristics except for gender, F (2, 112) = 3.75, *p* < 0.05. Descriptive data show that there are more female residents at regular small-scale living facilities compared with green care farms and traditional nursing homes (87% vs. 68% and 62% respectively). Pairwise comparisons revealed significantly more female residents at regular small-scale living facilities compared with traditional nursing homes (*p* < 0.05).

**Table 1:** Background characteristics, outcome indicators, and structure indicators

Background characteristics (range)	Total (*N* = 115)		Green care farm (*N* = 34)		Traditional nursing home (*N* = 29)		Regular small- scale living facility (*N* = 52)		*p*-value
	M	SD	M	SD	M	SD	M	SD	
Age (59–97)	83.8	7.8	82.1	8.5	82.6	8.3	85.5	6.8	0.1
Gender (% female)	75		68		62		87		0.03
Barthel index (0–20)	9.7	5.9	9.1	5.7	9.4	6.6	10.3	5.7	0.6
S-MMSE (0–30)	8.4	6.8	8.1	6.7	7.5	7	9.1	6.9	0.6
Outcome indicators	Total		Green care farm		Traditional nursing home		Regular small- scale living facility		*p*-value
	*N*	%	*N*	%	*N*	%	*N*	%	
Malnutrition	12	10	5	15	5	17	2	4	0.1
Missing	7	6	1	3	2	7	4	7	
Physical restraints	13	11	1	3	5	17	7	13	0.1
Missing	6	5	1	3	1	3	4	8	
Pressure ulcers	7	6	4	12	0	0	3	6	0.2
Missing	5	4	0	0	0	0	5	9	
Falling incidents	16	14	6	18	3	10	7	13	0.7
Missing	1	1	0	0	0	0	1	2	
Medication incidents	4	4	0	0	1	3	3	6	0.4
Missing	7	6	1	3	0	0	6	12	
Antipsychotic use	22	19	8	23	6	21	8	15	0.6
Missing	0	0	0	0	0	0	0	0	
Structure indicators	Total		Green care farm		Traditional nursing home		Regular small- scale living facility		
Total hours per resident per day (HPRD)*	3.1		3.1		3.1		3.2		
HPRD nurse assistant staffing/nurse aid staffing*	1		1.3		1.1		0.7		
HPRD Certified nurse assistant staffing*	1.8		1.5		1.5		2.4		
HPRD Vocationally trained RN staffing*	0.3		0.4		0.4		0.1		
HPRD baccalaureate-educated RN staffing*	0		0		0		0		

* Nightshift excluded

### Quality of care

#### Outcome indicators

Table 1 shows the scores on each of the measured outcome indicators. Descriptive statistics showed that overall; residents scored similar on all outcome indicators, regardless of nursing home type. Fisher’s exact test revealed no significant differences between green care farms, traditional nursing homes and regular small-scale living facilities.

#### Structure indicators

Table 1 shows the hours per resident per day (HPRD) for each type of nursing home. The figures were similar for green care farms, traditional nursing homes and regular small-scale living facilities. Data suggested that there were some minor differences between the educational levels of the staff in the different types of nursing homes. In descriptive terms there were more nurse assistants/nurse aids (less than 2 year education) and vocationally trained registered nurses (RNs) (at least 4 year education) at green care farms and traditional nursing homes than in regular small-scale residential facilities, where there were more certified nurse assistants (2–3 year education).

#### Process indicators

Table 2 provides information regarding the content of protocols. All nursing homes had protocols covering quality improvement, staff deployment, client records, physical restraints, and medication safety. All protocols were periodically evaluated and updated if necessary. All nursing home facilities were part of, or cooperated with three larger care organizations based in the Netherlands. This means that some of the participating facilities were influenced by the same organizational rules and board of directors. The care organizations had an electronic portal through which all staff had access to the protocols. All staff was notified when protocols were added or revised via a communication folder or an organizational newsletter. The content of the protocols was similar across settings. Quality improvement protocols included provisions for client satisfaction surveys, complaint commissions, client boards, incident registration and quality labels. All nursing homes had guidelines covering supervision of residents. Client records consisted of a paper record and an electronic record containing a care plan, daily reports and client-related agreements. All the physical restraints protocols were designed to minimize physical restraints. Medication safety protocols were also similar across settings.

**Table 2:** Content of protocols regarding care delivery

Content of protocols	
Quality improvement	Several activities aimed at quality improvement are described. First, all nursing homes work with a client satisfaction survey which is spread out periodically to all first responsible informal caregivers of the residents. Second, mechanisms for handling complaints are installed by means of a ‘complaint commission’ and a client board. Third, all incidents are registered in a national reporting system which is checked by the Health Care Inspectorate. Fourth, participating nursing homes had a nationally recognized quality label which was granted after inspection.
Staff employment	All nursing homes had specified in their protocols that nursing home care does not mean residents need to be supervised or watched 24 h every day. Instead, nursing homes use the term ‘attentive supervision’, which means that each resident is supervised at the level that he or she needs. A general guideline is that residents are left ‘unsupervised’ for a maximum of 10 minutes. Furthermore, all participating nursing homes could use technological aids if this is deemed necessary.
Client records	Each client has its’ own personal record consisting of a paper record and an electronic one. In the paper record a care agreement and the indication for a care package are included. The electronic record contains a care plan, daily reports, and client-related agreements. The client records need to be complete 6 weeks after admission (meaning it should also be evaluated by family members of the resident). Each half year the client records are discussed within a multidisciplinary team and if needed adjusted.
Physical restraints	All participating nursing homes try to reduce the physical restraints to a minimum. The goal is to look for alternatives for physical restraints. However, the guidelines for these alternatives differ across settings. At green care farms, there were no defined guidelines on which alternatives to use when. At the other types of nursing homes it was clearly stated when to use a particular alternative for physical restraints. In addition, it was determined which staff was allowed to apply certain measures. At green care farms, this was left unspecified.
Medication safety	For medication safety, no differences were found between green care farms and other types of nursing homes. All nursing homes use individual and up to date medication lists. Medication is wrapped in individual doses which are kept in a medication cabinet. Only the responsible physicians are allowed to alter medication prescriptions.

### Quality of life

Table 3 shows the descriptive results of the scores related to quality of life. The results of the multilevel regression analyses are shown in Table 4. Self-reports of the QoL-AD did not differ significantly between green care farms and the other types of nursing homes, however, they did indicate a meaningful difference (3 or more points) between residents of green care farms and residents of traditional nursing homes. Results of the proxy-reports were in the same direction and did reach significance (*p* < 0.05, ES = 0.8), suggesting that residents of green care farms had a better quality of life compared with residents of traditional nursing homes. In line with these findings, residents of green care farms scored higher than residents of traditional nursing homes on three Qualidem domains: positive affect, social relations and having something to do (*p* < 0.05, ES > 0.7). Table 4 shows that overall, residents of green care farms and regular small-scale living facilities had similar scores on quality of life and related outcomes. No differences were found between green care farms and regular small-scale living facilities.

**Table 3:** Quality of life and related outcomes

Quality of life and related outcomes (range)	Total (*N* = 115)		Green care farm (*N* = 34)		Traditional NH (*N* = 29)		Regular SSL (*N* = 52)	
	M	SD	M	SD	M	SD	M	SD
QoL-AD proxy report (13–52)	31.7	5	32.9*	4.5	29.1*	4.9	32.5	4.9
QoL-AD self-report (13–52)	37.3 (*n* = 66)	4.7	37.6 (*n* = 21)	4.1	35.2 (*n* = 15)	6	38.2 (*n* = 30)	4.2
Qualidem								
Care relationship (0–21)	15.1	4.6	16	4.9	14.9	4.2	14.6	4.6
Possitive affect (0–18)	14.1	3.7	15.8*	3.6	12.9*	3.5	13.8	3.6
Negative affect (0–9)	6	2.2	6	2.6	6.7	2.1	5.6	2.1
restless tense behavior (0–9)	5.4	2.9	5.2	2.7	5.5	2.8	5.5	3
positive self-image (0–9)	7.1	1.9	7.3	2.1	7.8	1.6	6.6	2
social relations (0–18)	12	3.7	13*	3.5	10.4*	3.8	12.3	3.6
Social isolation (0–9)	6.5	2.2	6.9	2.4	6.7	1.8	6	2.3
feeling at home (0–12)	9.6	2.5	9.5	2.9	9.9	2.2	9.4	2.3
having something to do (0–6)	2.7	2	3*	2.2	1.6*	1.8	3	1.9
RISE (0–6)	4.1	2	4.4	1.9	3.4	1.8	4.4	2
NPI-NH (0–144)	15.9	15.7	17.3	17.5	18.6	14	13.6	15.3
CMAI (29–203)	41.9	12.5	41.5	12.2	42.4	11.3	41.8	13.5
CSDD (0–38)	5.4	4.9	5	4.4	6.4	5.4	5	4.9

* Significant difference at α = .05

Underlined scores represent favorable scores

**Table 4:** Random-effects regression analysis on quality of life and related outcomes controlling for age, gender, cognition (S-MMSE), and independence in activities of daily living (Barthel index)

QoL-AD proxy report	ICC = 0.21	B	Std. Error B	95% confidence interval		Variance	*P* value	Effect size
Traditional nursing home		−3.7	1.7	−7.2	−.07	19	0.046*	0.8
Regular small-scale living facility		−1.2	1.4	−4.2	1.9	19	0.4	0.3
QoL-AD self-report	ICC = 0.14	B	Std. Error B	95% confidence interval		Variance	*P* value	Effect size
Traditional nursing home		−2.3	1.9	−6.5	1.9	21	0.3	0.5
Regular small-scale living facility		.09	1.6	−3.4	3.6	21	0.9	0.02
Qualidem Care relationship	ICC = 0.07	B	Std. Error B	95% confidence interval			*P* value	Effect size
Traditional nursing home		−.9	1.4	−3.9	2.1	20	0.5	0.2
Regular small-scale living facility		−1	1.2	−3.5	1.6	20	0.4	0.2
Qualidem Possitive affect	ICC = 0.12	B	Std. Error B	95% confidence interval			*P* value	Effect size
Traditional nursing home		−2.8	1.2	−5.4	−.2	13	0.037*	0.7
Regular small-scale living facility		−1.9	1	4.1	.4	13	0.1	0.5
Qualidem Negative affect	ICC = 0.09	B	Std. Error B	95% confidence interval			*P* value	Effect size
Traditional nursing home		.7	.7	−.7	2.1	4	0.3	0.4
Regular small-scale living facility		−.05	.6	−1.3	1.2	4	0.9	0.3
Qualidem restless tense behavior	ICC = 0.15	B	Std. Error B	95% confidence interval			*P* value	Effect size
Traditional nursing home		.3	1	−1.7	2.4	7	0.7	0.1
Regular small-scale living facility		.1	.8	−1.6	1.9	7	0.9	0.03
Qualidem positive self-image	ICC = 0.15	B	Std. Error B	95% confidence interval			*P* value	Effect size
Traditional nursing home		.5	.6	−.9	1.9	3	0.4	0.3
Regular small-scale living facility		−.3	.5	−1.4	.9	3	0.6	0.2
Qualidem social relations	ICC = 0.13	B	Std. Error B	95% confidence interval			*P* value	Effect size
Traditional nursing home		−2.5	1.1	−4.8	−.1	10	0.042*	0.8
Regular small-scale living facility		−1.3	.9	−3.3	.7	10	0.2	0.4
Qualidem Social isolation	ICC = 0.19	B	Std. Error B	95% confidence interval			*P* value	Effect size
Traditional nursing home		−.1	.8	−1.9	1.6	4	0.9	0.1
Regular small-scale living facility		−.8	.7	−2.3	.7	4	0.3	0.4
Qualidem feeling at home	ICC = 0.13	B	Std. Error B	95% confidence interval			*P* value	Effect size
Traditional nursing home		.5	.8	−1.2	2.2	5	0.5	0.2
Regular small-scale living facility		.3	.7	−1.1	1.8	5	0.6	0.1
Qualidem having something to do	ICC = 0.16	B	Std. Error B	95% confidence interval			*P* value	Effect size
Traditional nursing home		−1.3	.6	−2.6	−.1	2	0.035*	0.9
Regular small-scale living facility		−.3	.5	−1.3	.8	2	0.6	0.2
RISE	ICC = 0.13	B	Std. Error B	95% confidence interval			*P* value	Effect size
Traditional nursing home		−.9	.6	−2.2	.3	3	0.1	0.5
Regular small-scale living facility		−.3	.5	−1.4	.8	3	0.6	0.2
NPI-NH	ICC = 0.08	B	Std. Error B	95% confidence interval			*P* value	Effect size
Traditional nursing home		1.4	4.7	−8.9	11.6	230	0.8	0.3
Regular small-scale living facility		−2.3	4.2	−11.1	6.5	230	0.6	0.2
CMAI	ICC = 0.21	B	Std. Error B	95% confidence interval			*P* value	Effect size
Traditional nursing home		.9	4.7	−9.3	11	153	0.9	0.1
Regular small-scale living facility		1.3	4.1	−7.4	9.9	153	0.8	0.1
CSDD	ICC = 0.11	B	Std. Error B	95% confidence interval			*P* value	Effect size
Traditional nursing home		.8	1.5	−2.5	4.1	22	0.6	0.2
Regular small-scale living facility		.1	1.3	−2.8	2.9	22	0.96	0.02

## Discussion

The findings of this study indicate that overall quality of care at green care farms is comparable with quality of care at regular small-scale living facilities and traditional nursing homes. Similar scores were found on outcome indicators such as falling incidents and pressure ulcers. In addition, the hours per resident per day did not differ across settings. Lastly, all types of nursing homes had comparable protocols present, and accessibility and content of the protocols differed minimally. Looking at quality of life and related outcomes, some findings suggest that residents of green care farms had a better quality of life than residents of traditional nursing homes.

Limitations of the study must be acknowledged: the cross-sectional design, lack of randomization and the fairly small sample size. The cross-sectional design means that we could not determine the causal relationships between type of nursing home and the outcome variables. Ideally there should be a large, randomized controlled study of newly admitted residents covering more nursing homes and following residents over a longer period of time. Although this kind of study would produce more generalizable findings, it has several ethical and practical drawbacks. Randomization of residents to different types of nursing homes is not feasible, as people with dementia and their family caregivers are free to go to the nursing home of their choice. Furthermore, there are still very few green care farms providing 24-h care, making it impossible to include more green care farm residents. In addition, another limitation has to do with the way data was collected. Green care farms, regular small-scale living facilities and traditional nursing homes all operate within the same health system, with the same legislation, funding, and quality assurance systems (including health care inspectorate visits) [14]. This study shows that all three types of nursing homes adhere to these regulations. However, information gathered in this study on process indicators of quality of care might not paint a reliable picture on the actual care processes at the different types of nursing homes. Future studies should also focus on whether care process guidelines are adhered to in daily care practice by for instance performing observations or conducting interviews with formal caregivers.

The current study suggests a better quality of life at green care farms compared with traditional nursing homes. Difference in the daily life of residents at these different types of nursing homes may influence their quality of life. Previous research indicates that people with dementia at green care farms were more engaged in activities and had more social interaction than people with dementia at regular care facilities, both in day-care and in nursing home care [16, 31]. Being engaged in activities, and having social interactions are both important factors influencing quality of life [7, 32]. The green care farms included in this study focus on the remaining capabilities of people with dementia and try to use them as the basis for tailored activities that are integrated into normal daily care. For instance, residents who are still mobile are asked to help with milking the cows and residents who are still able to cook are asked to help with preparing dinner.

Another factor which may explain why residents at green care farms have a better quality of life is the physical environment. The integration of activities and care may be enhanced because the physical environment of green care farms includes, for instance, open doors, large outdoor freely accessible spaces, gardens and stables. Several studies have indicated the importance of the physical environment to people with dementia [32, 33]. Beerens and colleagues (2016) showed that residents who frequently participated in outdoor activities had higher mood scores than residents who went outdoors less often. Furthermore, several reviews indicate that factors such as privacy, autonomy, view, nature, orientation, safety and domesticity are important aspects of the physical environment and can have an effect on people with dementia [33]. It is plausible that green care farms have a physical environment that has a large potential to be beneficial for their residents.

However, just making changes to the physical environment of a nursing home is not sufficient to obtain the potential beneficial effects. Nursing staff play an important role in ensuring that the physical environment of a nursing home is used to its full potential. We found no difference in the staff-resident ratio across the different types of nursing homes. Our results suggest that differences in quality of life cannot be explained by staffing levels. There is evidence that improving quantitative measures of staffing is not sufficient to improve care quality; the quality of staff is also important [7, 34]. Within green care farms and regular small-scale living facilities, care staff has different roles and tasks compared with traditional nursing homes [3]. Even though many existing nursing homes are moving towards more focus on person-centred care, and engaging people with dementia in meaningful activities, this remains a struggle for many nursing homes. Future research should focus on how we can implement successful factors of innovative nursing homes into other settings by for instance studying on how to integrate the different tasks of staff.

Despite unique features in the physical and organizational environment of green care farms, the current study did not find any differences between green care farms and regular small-scale living facilities regarding quality of life and quality of care. This makes sense, considering they provide care according to the same underlying psychosocial care concept with normalization as an important guiding principle [3]. Although a previous study did found differences on the amount of physical activity of residents of green care farms and regular small-scale living facilities [16], the differences in the environment are not translated into quality of life related outcomes.

Our findings have some implications for practice and education. There should be more focus on determining which staff competences are positively associated with care quality and residents’ quality of life in nursing homes. There is an increased interest in the concept of psychosocial care, which emphasizes e.g. person-centred care and provision of meaningful activities [3, 4]. However, in practice this remains difficult to achieve. More attention should be paid to finding ways for nursing staff to integrate activities into daily care practices and matching everyday activities to individual residents’ preferences and needs. Lastly, it is important to keep different perspectives in mind. This study shows that people with dementia score their quality of life differently than proxies do. Previous research also shows that people with dementia and caregivers do not consider the same domains to be important for their quality of life [35]. It is important that the perspective of the people with dementia remains the starting point for providing person-centred care. Qualitative studies on the experiences of people with dementia and their informal caregivers can be beneficial for exploring their perspectives on the care provided at different types of nursing homes.

## Conclusions

In conclusion this study shows that green care farms are a valuable alternative to existing nursing homes. This is important as people with dementia are a heterogeneous group with varying needs. In order to provide tailored care there also is a need for a variety of living environments.
